# Association between pathologic chemotherapy response score and pattern of recurrence in advanced high-grade serous ovarian cancer

**DOI:** 10.1093/oncolo/oyag055

**Published:** 2026-03-30

**Authors:** Francesco Mezzapesa, Antonio De Leo, Camelia Alexandra Coada, Elisabetta Pia Bilancia, Lucia Genovesi, Giulia Mantovani, Caterina Ravaioli, Daniela Turchetti, Dario De Biase, Daniela Rubino, Claudio Zamagni, Pierandrea De Iaco, Anna Myriam Perrone

**Affiliations:** Division of Oncologic Gynecology, IRCCS Azienda Ospedaliero-Universitaria di Bologna, 40138, Bologna, Italy; Department of Medical and Surgical Sciences (DIMEC), University of Bologna, Bologna, Italy; Department of Medical and Surgical Sciences (DIMEC), University of Bologna, Bologna, Italy; Solid Tumor Molecular Pathology Laboratory, IRCCS Azienda Ospedaliero-Universitaria di Bologna, 40138, Bologna, Italy; Department of Morpho-functional sciences, “Iuliu Hațieganu” University of Medicine and Pharmacy, Cluj-Napoca, Romania; Division of Oncologic Gynecology, IRCCS Azienda Ospedaliero-Universitaria di Bologna, 40138, Bologna, Italy; Department of Medical and Surgical Sciences (DIMEC), University of Bologna, Bologna, Italy; Division of Oncologic Gynecology, IRCCS Azienda Ospedaliero-Universitaria di Bologna, 40138, Bologna, Italy; Department of Medical and Surgical Sciences (DIMEC), University of Bologna, Bologna, Italy; Division of Oncologic Gynecology, IRCCS Azienda Ospedaliero-Universitaria di Bologna, 40138, Bologna, Italy; Department of Medical and Surgical Sciences (DIMEC), University of Bologna, Bologna, Italy; Solid Tumor Molecular Pathology Laboratory, IRCCS Azienda Ospedaliero-Universitaria di Bologna, 40138, Bologna, Italy; Department of Medical and Surgical Sciences (DIMEC), University of Bologna, Bologna, Italy; Medical Genetics Unit, IRCCS Azienda Ospedaliero-Universitaria Di Bologna, Bologna, Italy; Solid Tumor Molecular Pathology Laboratory, IRCCS Azienda Ospedaliero-Universitaria di Bologna, 40138, Bologna, Italy; Department of Pharmacy and Biotechnology, University of Bologna, Bologna, Italy; Addarii Medical Oncology, IRCCS Azienda Ospedaliero-Universitaria Di Bologna, Bologna, Italy; Addarii Medical Oncology, IRCCS Azienda Ospedaliero-Universitaria Di Bologna, Bologna, Italy; Division of Oncologic Gynecology, IRCCS Azienda Ospedaliero-Universitaria di Bologna, 40138, Bologna, Italy; Department of Medical and Surgical Sciences (DIMEC), University of Bologna, Bologna, Italy; Division of Oncologic Gynecology, IRCCS Azienda Ospedaliero-Universitaria di Bologna, 40138, Bologna, Italy; Department of Medical and Surgical Sciences (DIMEC), University of Bologna, Bologna, Italy

**Keywords:** neoadjuvant chemotherapy, CRS, oligometastasis, ovarian cancer, PARPi

## Abstract

**Background:**

Chemotherapy Response Score (CRS) is a three-tier histopathologic system evaluating response to neoadjuvant chemotherapy (NACT) in high-grade serous ovarian cancer (HGSOC).

**Methods:**

We evaluated recurrence patterns and survival outcomes in advanced-stage HGSOC patients treated with NACT and interval debulking surgery (IDS) between 2015 and 2024.

**Results:**

Among 238 patients, CRS3 was most frequent (43.3%) and was associated higher complete cytoreduction (CC0) rates (93%) compared to CRS2 (81%) and CRS1 (57%) (*p* < 0.001). Median follow-up was 35 months (IQR 12.6–42.2). Median progression-free survival (PFS) was 40.4 months for CRS3, 23.4 for CRS2, and 21.5 for CRS1 (*p* = 0.001). Among 109 (45.7%) patients who relapsed, 25 (23%) presented with oligometastatic disease (≤5 lesions), more frequently in CRS3 (46%) than CRS2 (8.9%) or CRS1 (15%) (*p* < 0.001). Specifically, peritoneal (*p* = 0.002) and nodal (*p* = 0.05) oligorecurrences were more common in the CRS3 group. CRS3 predicted oligometastatic recurrence (OR = 4.89; *p* = 0.008) and was associated with increased use of locoregional therapies (surgery or radiotherapy) at relapse (*p* = 0.014). Median overall survival (OS) was 93.9, 37.2, and 31.7 months for CRS3, CRS2, and CRS1, respectively (*p* < 0.001).

**Conclusions:**

CRS3 predicts lower recurrence risk, increased rates of oligometastatic relapse, and greater use of locoregional treatments in patients with recurrent HGSOC after NACT and IDS.

Implication for practice Chemotherapy Response Score 3 shows lower recurrence, more oligometastatic relapse, and higher locoregional therapy in patients with HGSOC.

## Introduction

High-grade serous ovarian cancer (HGSOC) is the most common histologic subtype of epithelial ovarian cancer and is diagnosed at an advanced stage in up to 80% of cases^1^. Standard treatment typically involves cytoreductive surgery combined with platinum-based chemotherapy. In patients with a high tumor burden or poor performance status, surgery can be delayed after 3–4 cycles of neoadjuvant chemotherapy (NACT) as a valid alternative to primary debulking surgery (PDS).^2^ However, emerging evidence suggests that extending NACT to six or more cycles may be an effective strategy to increase the likelihood of achieving complete cytoreduction (CC0).^3^^,^^4^

Despite high initial response rates to platinum-based therapy, most patients eventually relapse. Approximately 60–75% of recurrences manifest as multifocal peritoneal carcinomatosis, whereas a smaller proportion presents with oligometastatic disease,^5^^,^^6^ defined as five or fewer metastatic lesions.^7^ This latter pattern is associated with improved survival outcomes,^5^ as it may be amenable to localized approaches such as secondary debulking surgery (SDS) or stereotactic radiotherapy.^8^ The lack of validated tools to predict recurrence patterns limits personalized post-treatment approaches.

Previous studies suggested that NACT may promote selection of platinum-resistant clones with aggressive behavior, resulting in a higher incidence of multifocal peritoneal recurrence compared to PDS.^9^ However, outcomes after NACT are heterogeneous, with variable responses despite similar treatment^4^^,^^10^ likely due to underlying tumor biology.^11^

The Chemotherapy Response Score (CRS), introduced by Böhm et al. in 2015, is the earliest objective histopathological biomarker of treatment response in this setting.^12^ It is a three-tier grading system designed to assess tumor regression following NACT, originally in omental specimens and investigated on adnexal tissues.^13^ CRS1 indicates minimal/no response, CRS2 partial response, and CRS3 complete/near-complete tumor regression.^14^ CRS shows high interobserver reproducibility and is recommended for routine reporting in international guidelines.^15^ It is a validated marker of chemosensitivity and a strong prognostic factor for both progression-free and overall survival.^16^ However, its role in predicting anatomical patterns of recurrence remains unexplored.

This study aims to investigate the association between pathological response to NACT and the pattern of disease recurrence in patients with advanced HGSOC.

## Methods

### Study design and patients’ selection

This retrospective, observational, single-center study included patients with HGSOC who underwent platinum-based NACT followed by interval debulking surgery (IDS) between 2015 and 2024. The study was approved by the local Ethical Committee (AVEC: 524/2022/Oss/AOUBo). Eligible patients had histologically confirmed HGSOC, FIGO stage III or IV, and underwent NACT followed by IDS. Only cases with available CRS assessment on omental specimens were included. Exclusion criteria were non-serous histology, IDS not performed, IDS performed elsewhere, no CRS evaluation, prior or synchronous malignancies within five years (except basocellular or in situ tumors), and missing recurrence data.

### Data collection

Clinical, surgical, and pathological data were retrieved from the institutional database. All patients underwent thoraco-abdominal-pelvic computed tomography (CT), serum CA-125 measurement baseline. Diagnostic laparoscopy was routinely performed pre-NACT, repeated after 3–4 cycles, and subsequently whenever evaluation of operability was required. Disease burden was assessed by the Peritoneal Cancer Index (PCI) and Fagotti score,^17^^,^^18^ with histologic confirmation.^19^ Following multidisciplinary assessment, and considering patients’ performance status, women received between 3 and 6 cycles of platinum and paclitaxel-based NACT.^15^^,^^20^ Timing of IDS was based on performance status, CA125 kinetics, and radiologic response, as detailed previously.^21^ When surgery was feasible, longitudinal laparotomy was performed. In selected cases with low tumor burden after NACT, a minimally invasive approach was considered, based on intraoperative evaluation during diagnostic laparoscopy. Hyperthermic intraperitoneal chemotherapy (HIPEC) was administered within clinical trial.^22^

Pathological response was graded on omental specimens using Böhm’s chemotherapy response scoring^12^ system. These scores were recorded by dedicated pathologists as part of the standard institutional diagnostic protocol and included in the final reports at the time of the original pathological examination. and was included in the final pathology report The number of adjuvant chemotherapy cycles was determined by the number of prior neoadjuvant chemotherapy (NACT) cycles, up to a total maximum of nine as per local practice. Maintenance therapies were administered according to the international guidelines in effect at the time of treatment.^20^^,^^23^

Follow up was conducted according to international guidelines^24^ and local protocols, with routine clinical evaluations and CA-125 measurement every three months, thoraco-abdominal CT imaging every six months during the first three years and annually thereafter. Unscheduled visits were arranged upon symptom onset or patient request. Disease recurrence was assessed by an expert radiology team, and images were reviewed during multidisciplinary tumor board meetings. Recurrence patterns were classified as oligometastatic (≤5 lesions) or multifocal (>5 lesions), based on surgical findings when available, otherwise by radiological assessment. Sites of recurrence and treatment strategies were recorded. Patients with potentially resectable disease underwent laparoscopic assessment, followed by SDS when feasible. Stereotactic radiotherapy was considered for patients with solitary lesions not amenable to surgical resection, if deemed feasible by the radiotherapist. Patients who underwent surgery or radiotherapy were subsequently treated with systemic chemotherapy in accordance with standard guidelines and at the oncologist’s discretion. Patients with multifocal or unresectable disease were treated with systemic chemotherapy alone.

### Statistical analysis

Statistical analysis used Kolmogorov-Smirnov for normality. Continuous variables were expressed as mean ± *SD* or median (IQR); categorical as counts and percentages. Group comparisons used Kruskal-Wallis, Chi-square, or Fisher’s test. Post-hoc pairwise comparisons were conducted with Bonferroni correction. Logistic regression identified predictors of oligometastatic recurrence. Median follow-up time was estimated using the reverse Kaplan-Meier method. Progression-free survival (PFS) was defined as the time from the date of initial diagnosis to the date of documented disease progression or recurrence. Overall survival (OS) was defined as the time from diagnosis to either death from any cause or the last known date the patient was alive. Patients without progression or death at the time of last follow-up were censored in the survival analysis. Kaplan-Meier and Cox models were used for PFS and OS. Comparisons between groups were performed using the log-rank test. Analyses were conducted in R software, version 4.4.0 (2024-04-24 ucrt). Statistical significance was set at *p* ≤ 0.05.

## Results

Over the study period, 414 patients underwent NACT followed by IDS; after applying the inclusion criteria (Supplementary Figure 1), 238 (57.5%) patients were retained for the final analysis. Among these, 43 (18.1%) showed minimal or no histologic response (CRS1), 92 (38.6%) had a partial response (CRS2), and 103 (43.3%) achieved a complete or optimal response (CRS3). No differences were observed in median age at diagnosis (*p* = 0.170), baseline BMI (*p* = 0.967), FIGO stage (*p* = 0.925), baseline serum CA125 levels (*p* = 0.939), or BRCA mutation status (*p* = 0.642) (Table 1; Supplementary Table 1). The baseline Fagotti score was similar across all groups (*p* = 0.714). Median baseline PCI reflected a high disease burden (22, IQR 18–26), with higher values in the CRS1 group (*p* = 0.033).

**Table 1.** General characteristics of the patients included in the study.

**Variable**	CRS1	CRS2	CRS3	*p*-Value^2^
	*N* = 43^1^	*N* = 92^1^	*N* = 103^1^	
Age at diagnosis, years	61 (53, 69)	65 (58, 71)	63 (55, 71)	0.170
BRCA status				0.642
Wild Type	15 (60%)	46 (69%)	47 (55%)	
BRCA1 mut	6 (24%)	9 (13%)	22 (26%)	
BRCA2 mut	4 (16%)	10 (15%)	13 (15%)	
VUS	0 (0%)	2 (3.0%)	3 (3.5%)	
BRCA1/BRCA2 mut	0 (0%)	0 (0%)	1 (1.2%)	
Unknown	18	25	17	
HRD status				**0.029**
HRD negative	1 (50%)	8 (40%)	1 (6.3%)	
HRD positive	1 (50%)	12 (60%)	15 (94%)	
Unknown	41	72	87	
BMI (kg/m^2^)	23.8 (21.5, 31.3)	24.9 (21.6, 28.7)	24.3 (21.3, 27.7)	0.967
Baseline CA125 level (U/ml)	662 (332, 2.107)	984 (338, 1,897)	717 (340, 2,095)	0.939
Unknown	13	21	30	
Baseline PCI	25 (21, 28)	21 (16, 26)	21 (17, 25)	**0.033**
Unknown	17	38	53	
Baseline Fagotti Score				0.714
*≤* 8	19 (73%)	37 (69%)	32 (64%)	
> 8	7 (27%)	17 (31%)	18 (36%)	
Unknown	17	38	53	
FIGO Stage at diagnosis				0.925
III	33 (77%)	68 (74%)	76 (74%)	
IV	10 (23%)	24 (26%)	27 (26%)	
Number of NACT cycles	6.00 (3.00, 6.00)	6.00 (4.00, 6.00)	6.00 (3.00, 6.00)	0.849
PCI post NACT	13 (8, 20)	8 (3, 12)	0 (0, 3)	**<0.001**
Unknown	7	24	52	
Fagotti score post NACT				0.121
≤ 8	33 (92%)	64 (94%)	51 (100%)	
*>* 8	3 (8.3%)	4 (5.9%)	0 (0%)	
Type of IDS				**<0.001**
Minimally Invasive	4 (9.3%)	9 (9.8%)	32 (31%)	
Open	39 (91%)	83 (90%)	71 (69%)	
Surgery timing, minutes	180 (135, 225)	170 (150, 200)	155 (120, 210)	0.249
Unknown	13	59	68	
Aletti Complexity score	4.00 (2.00, 7.00)	4.00 (2.00, 5.00)	2.00 (2.00, 4.00)	**<0.001**
HIPEC use				0.406
yes	3 (7.0%)	14 (15%)	14 (14%)	
no	40 (93%)	78 (85%)	89 (86%)	
Residual Tumor				**<0.001**
CC0	24 (57%)	72 (81%)	93 (93%)	
≥CC1	18 (43%)	17 (19%)	7 (7.0%)	
Unknown	1	3	3	
Maintenance Therapy				**0.0023**
No Maintenance	14 (33%)	37 (40%)	28 (27%)	
ParpI	6 (14%)	33 (36%)	46 (45%)	
Bevacizumab	21 (49%)	21 (23%)	25 (24%)	
Others	2 (4.7%)	1 (1.1%)	4 (3.9%)	
Platinum Free Interval (PFI)				**0.021**
≤ 6 Months	4 (15%)	11 (25%)	1 (2.7%)	
> 6 Months	23 (85%)	34 (75%)	36 (97.3%)	

^1^Median (Q1, Q3); *n* (%);

^2^Kruskal-Wallis rank sum test; Fisher’s exact test; Pearson’s Chi-squared test; One-way analysis of means. CRS: Chemotherapy Response Score; IDS: Interval Debulking surgery; HIPEC: Hyperthermic Intraperitoneal Chemotherapy; CC0: Complete Citoreduction; CC1: Incomplete Cytoreduction; ParpI (Poly (ADP-ribose) polymerase inhibitors.#Bold values indicate statistically significant results (p < 0.05).

The median number of NACT cycles was 6 (IQR: 3–6) (*p* = 0.849), without different distribution of pathological response grade (Supplementaty Table 2). CRS3 patients had lower median Fagotti score post NACT (*p* < 0.001) and PCI post NACT scores (0, IQR: 0–3) compared to CRS2 (8, IQR: 3–12) and CRS1 (13, IQR: 8–20) (*p* < 0.001). CRS3 patients were significantly more likely to undergo minimally invasive surgery (*p* < 0.001) and required less extensive procedures (*p* < 0.001). CC0 was achieved in 93% of CRS3 patients, 81% of CRS2, and 57% of CRS1 (*p* < 0.001). HIPEC was administered in 31 patients (13%) without differences among groups (*p* = 0.406).

The median number of adjuvant chemotherapy cycles was 3 (IQR: 2–4), with no difference between groups (*p* = 0.234). HRD status, available in 15.9% of the cohort, was more frequent in CRS3 (*p* = 0.029). Maintenance therapy was given to 159 patients (67%). PARPi was used in 45% of CRS3, 36% of CRS2, and 14% of CRS1 patients. Conversely, bevacizumab was used in 49% of CRS1, compared to 24% and 23% in CRS2 and CRS3, respectively (*p* = 0.002).

### Pattern of recurrence

During a median follow-up of 35.0 months (95% CI, 30.3–42.1), 109 patients (45.7%) had disease recurrence: 27 (63%) in CRS1, 45 (49%) in CRS2, and 37 (36%) in CRS3 (*p* = 0.009). Patients with CRS3 demonstrated the highest rate of platinum-sensitive recurrence (Platinum Free Interval PFI > 6 months; 97.3%) compared to CRS1 and CRS2 patients (*p* = 0.021). Among them, 84 (77%) developed multifocal disease, while 25 (23%) had oligometastatic recurrence involving peritoneal, lymphatic, or parenchymal sites (Table 2; Supplementary Table 1). Oligometastatic recurrence was more frequent in CRS3 (46%) versus CRS2 (8.9%) and CRS1 (15%) (*p* < 0.001) Notably, the pattern of recurrence also differed significantly based on platinum sensitivity. All patients with platinum-resistant disease presented with multifocal recurrence, with only one exception. Conversely, among platinum-sensitive patients, an higher proportion presented with oligometastatic recurrence (24.8%).

**Table 2.** Patterns and anatomical sites of relapse.

Variable	CRS1	CRS2	CRS3	*p*-Value^2^
	*N* = 27^1^	*N* = 45^1^	*N* = 37^1^	
Type of recurrence (any site)				**<0.001**
Multifocal (>5 lesions)	23 (85%)	41 (91%)	20 (54%)	
Oligometastatic (≤5 lesions)	4 (15%)	4 (8.9%)	17 (46%)	
Number of sites for Oligometastatic lesions				0.089
1 lesion	0 (0%)	3 (75%)	9 (53%)	
2 lesions	2 (50%)	0 (0%)	3 (18%)	
3 lesions	0 (0%)	1 (25%)	0 (0%)	
4 lesions	0 (0%)	0 (0%)	4 (24%)	
5 lesions	2 (50%)	0 (0%)	1 (5.9%)	
Peritoneal Recurrence				**0.002**
Multifocal (>5 lesions)	21 (78%)	36 (80%)	15 (41%)	
Oligometastatic (≤5 lesions)	1 (3.7%)	3 (6.7%)	7 (19%)	
No lesions	5 (19%)	6 (13%)	15 (41%)	
Node Recurrence				**0.050**
Multifocal (>5 lesions)	7 (26%)	19 (42%)	10 (27%)	
Oligometastatic (≤5 lesions)	1 (3.7%)	1 (2.2%)	7 (19%)	
No lesions	19 (70%)	25 (56%)	20 (54%)	
Parenchymal Recurrence				0.358
Multifocal (>5 lesions)	3 (11%)	7 (16%)	5 (14%)	
Oligometastatic (≤5 lesions)	2 (7.4%)	0 (0%)	3 (8.1%)	
No lesions	22 (81%)	38 (84%)	29 (78%)	
Parenchymal Recurrence (site specific)				0.680
Liver	3 (60%)	3 (43%)	3 (38%)	
Lung	1 (20%)	3 (43%)	4 (50%)	
Liver and spleen	1 (20%)	0 (0%)	0 (0%)	
Brain	0 (0%)	0 (0%)	1 (13%)	
Osseous	0 (0%)	1 (14%)	0 (0%)	
Treatment strategy at recurrence				**0.014**
Chemotherapy alone	26 (96.3%)	43 (95.6%)	25(67.5%)	
Chemotherapy + Radiotherapy	0 (0%)	0 (0%)	4 (10.8%)	
Chemotherapy + Surgery	1 (3.7%)	2 (4.4%)	8 (21.6%)	

^1^Median (Q1, Q3); *n* (%);

^2^Kruskal-Wallis rank sum test; Fisher’s exact test; Pearson’s Chi-squared test; One-way analysis of means; CRS: Chemotherapy Response Score.#Bold values indicate statistically significant variables (p < 0.05).

**Figure 1.**
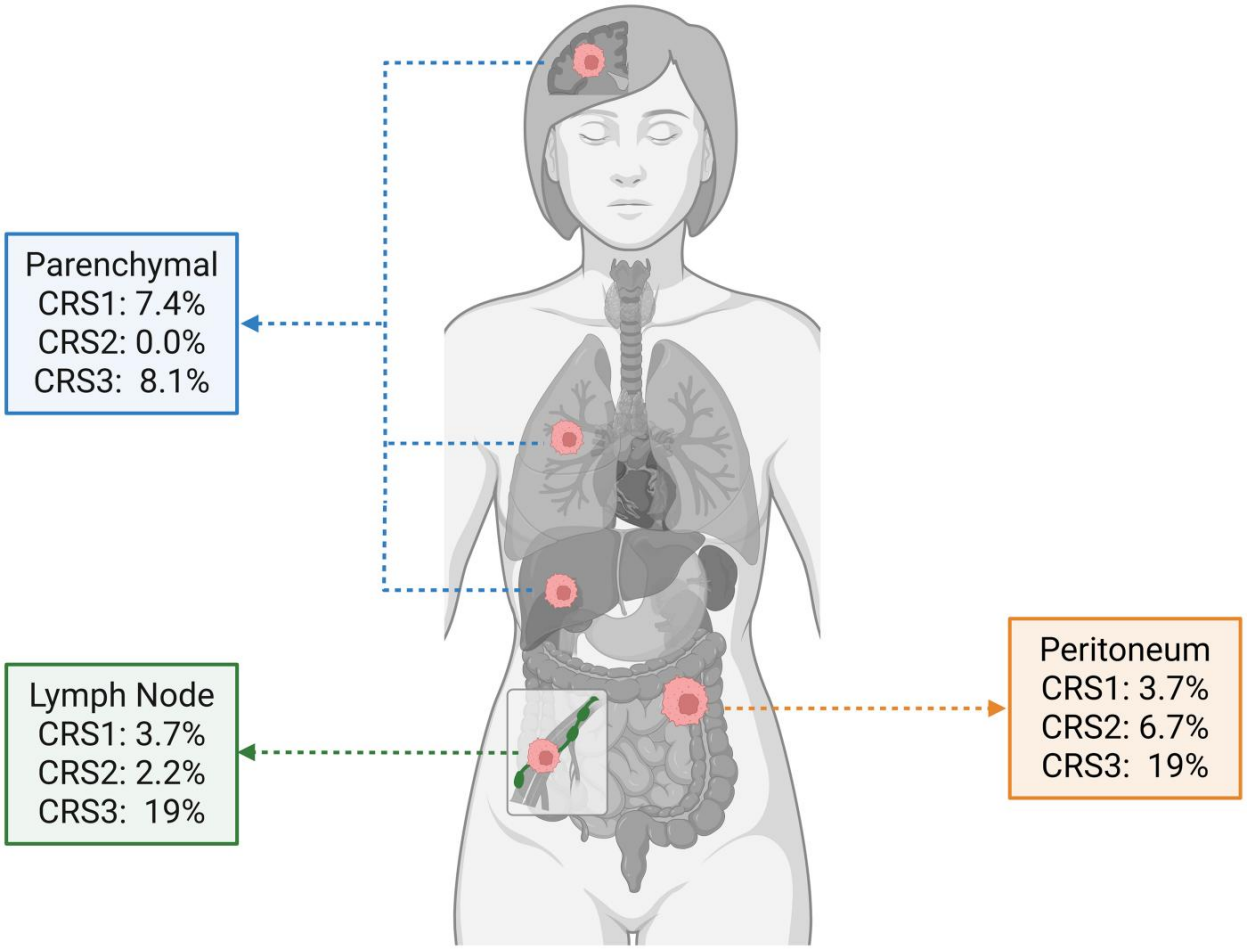
Distribution of oligometastatic recurrences according to CRS score.

Regarding metastasis site (Figure 1), multifocal peritoneal carcinomatosis occurred in most CRS1 (78%) and CRS2 (80%) cases, versus 41% in CRS3 (*p* < 0.001). Lymph node oligometastases were found in 3.7% of CRS1, 2.2% of CRS2, and 19% of CRS3 (*p* = 0.05). No significant differences emerged in parenchymal metastases (*p* = 0.358); the liver was most commonly involved (45%), followed by the lungs (8%). CRS3 patients were 5.18 times more likely to undergo surgery and 19.2 times more likely to receive radiotherapy, compared to CRS1 and CRS2, who were mainly treated with chemotherapy alone (96.3% and 95.6%, *p* = 0.014).

Logistic regression identified predictors of oligometastatic recurrence (Table 3). Variables for the univariate analysis were selected based on prior literature^25–27^ and included: BRCA status (OR = 1.49; 95% CI, 0.52–4.14), residual tumor (OR = 3.01; 95% CI, 0.78–19.89), HIPEC use (OR = 1.14; 95% CI, 0.3–3.68) CRS3 score (OR = 4.89; 95% CI, 1.52–19.19), maintenance therapy (OR = 2.54; 95% CI, 0.68–12.4) and stage at diagnosis (OR = 2.12; 95% CI, 0.71–6.01). CRS3 was the only variable significantly associated with oligometastatic recurrence. Multivariate analysis was not performed to avoid overfitting. Prospective validation in larger datasets is needed.

**Table 3.** Logistic regression for oligometastatic pattern of recurrence.

Variable	Univariate		
	OR	CI (95%)	*p*-Value
BRCA status			
BRCA wild type/VUS	1		
BRCA mutated	1.49	(0.52–4.14)	0.448
CRS			
CRS1	1		
CRS2	0.56	(0.12–2.57)	0.443
CRS3	4.89	(1.52–19.19)	**0.012**
HIPEC			
HIPEC No	1		
HIPEC Yes	1.14	(0.3–3.68)	0.832
Residual tumor			
≥CC1	1		
CC0	3.01	(0.78–19.89)	0.161
Maintenance Therapy			
No Maintenance	1		
Bevacizumab	1.78	(0.48–8.6)	0.416
ParpI	2.54	(0.68–12.4)	0.196
Others	0	(NA–>1e + 10)	0.991
Stage at diagnosis			
III	1		
IV	2.12	(0.71–6.01)	0.161

OR: Odds Ratio; CI: Confidence Interval; VUS: Variant of Unknown Significance; CRS: Chemotherapy Response Score; CC0: Complete Citoreduction; CC1: Incomplete Cytoreduction; ParpI (Poly (ADP-ribose) polymerase inhibitors.#Bold values indicate statistically significant variables (p < 0.05)

### Survival outcomes

**Figure 2.**
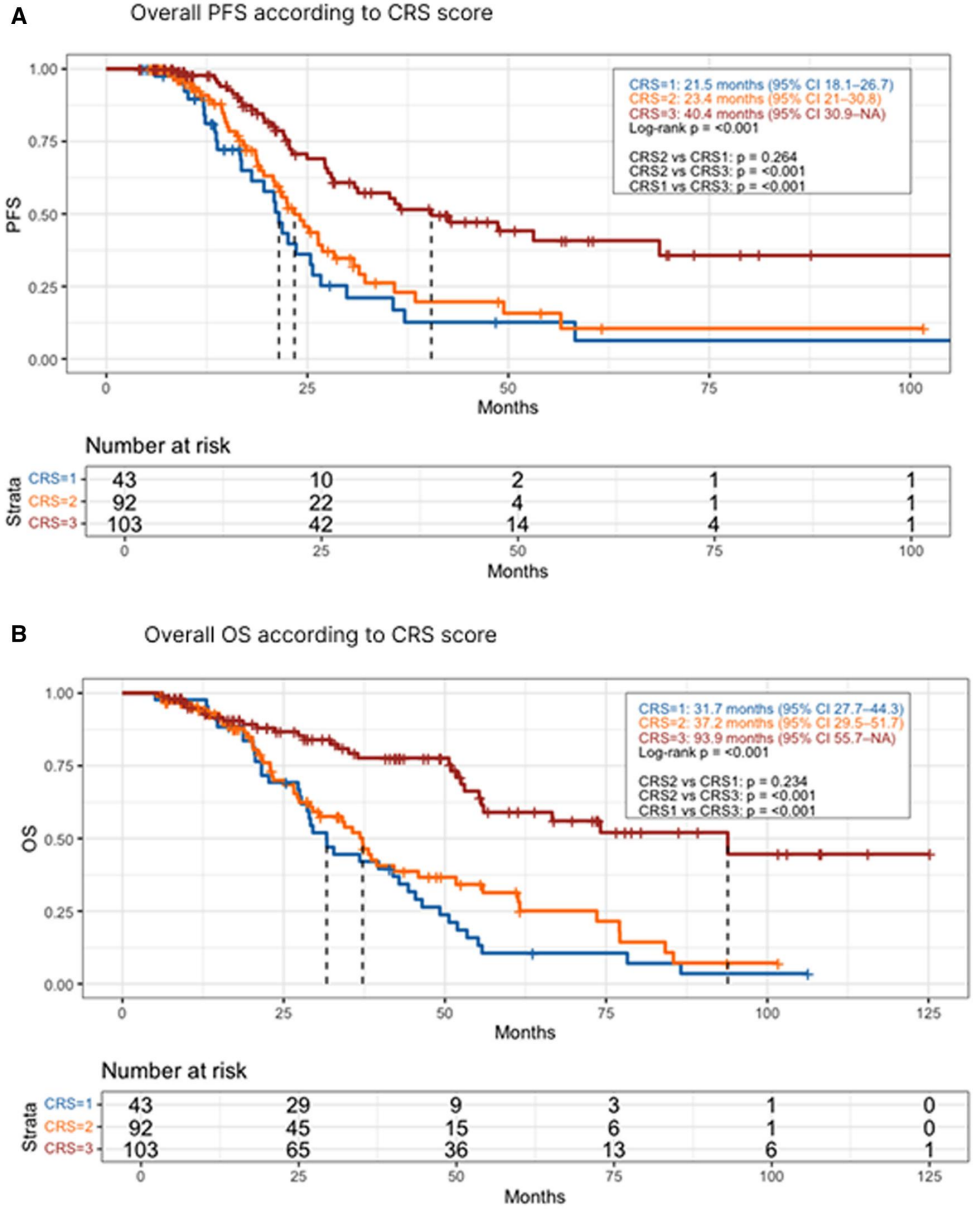
Kaplan-Meier curves. (A) Overall Progression Free Survival according to CRS score; (B) Overall Survival according to CRS score.

Median follow-up was 35.0 months (95%CI 30.3–42.1) (Supplementary Figure 2). CRS3 patients demonstrated significantly longer median PFS compared to CRS2 and CRS1 (40.4 vs. 23.4 vs. 21.5 months; *p* = 0.001) (Figure 2).

**Figure 3.**
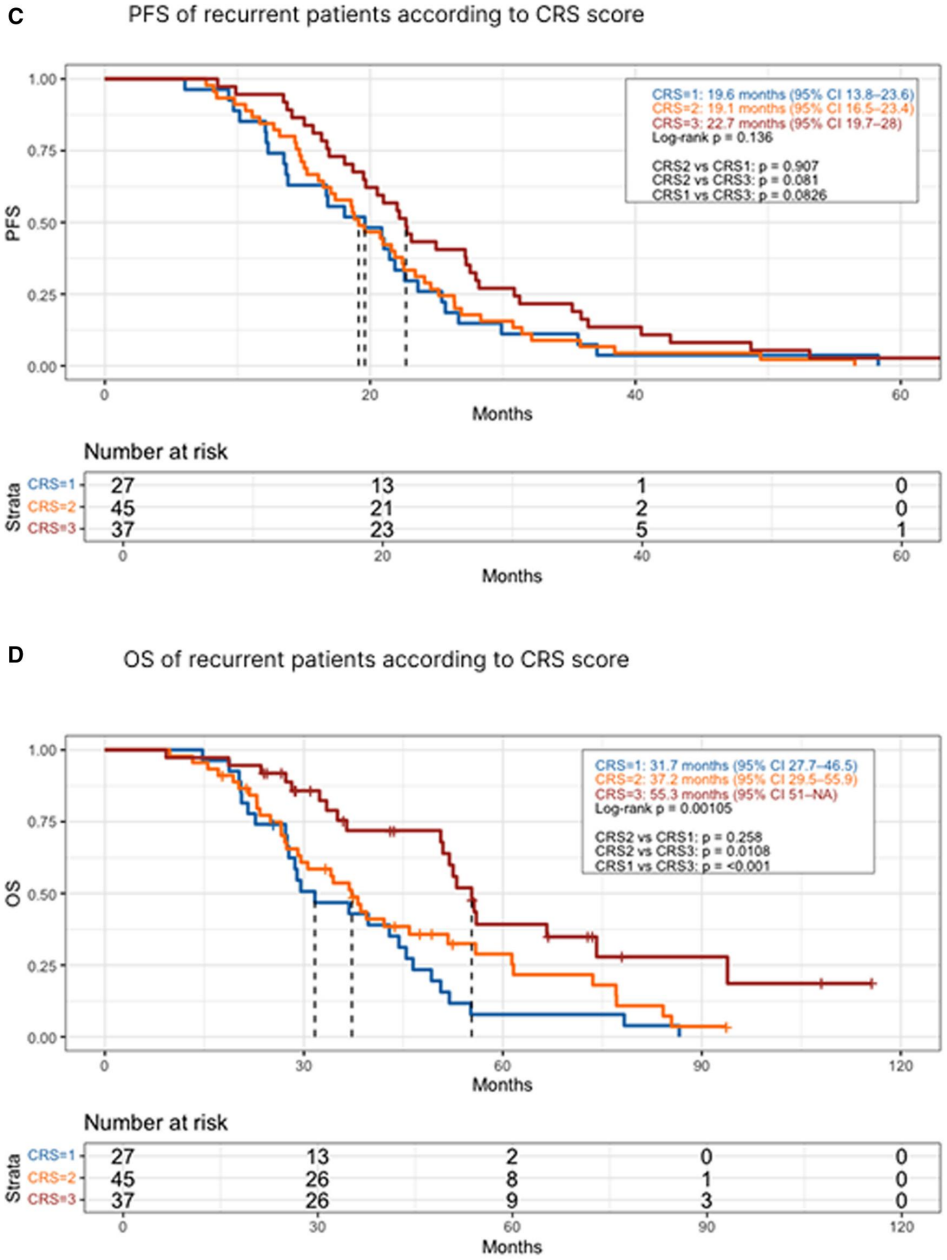
Kaplan-Meier curves. (C) Progression Free survival of recurrent patients according to CRS score; (D) Overall Survival of recurrent patients according to CRS score.

In univariate Cox regression analysis, age at diagnosis (HR = 1.01; 95%CI: 0.99–1.03), FIGO IV stage at diagnosis (HR = 0.7; 95%CI: 0.43–1.14), HIPEC use (HR = 1.27; 95%CI: 0.75–2.17), maintenance therapy with PARPi (HR = 0.86; 95%CI: 0.5–1.47) and oligometastatic recurrence (HR = 0.74; 95%CI: 0.47–1.16) were not statistically significant, while CRS3 response (HR = 0.34; 95%CI: 0.21–0.57), BRCA mutation (HR = 0.44; 95%CI: 0.28–0.69), and CC0 resection (HR = 0.50; 95%CI: 0.30–0.82) were significantly associated with a reduced risk of recurrence. In multivariate analysis, CRS3 response (HR = 0.44; 95%CI: 0.23–0.83), BRCA mutation (HR = 0.36; 95%CI: 0.22–0.59), and CC0 resection (HR = 0.29; 95%CI: 0.14–0.57) remained independent predictors (Table 4). In a sub analysis of only the patients who experienced recurrence, CRS3 patients still had longer PFS compared to CRS2 and CRS1 (22.7 vs. 19.1 vs. 19.6 months, respectively), although the difference was not statistically significant (*p* = 0.136) (Figure 3).

**Table 4.** Cox proportional hazards regression model for progression free survival and overall survival.

Variable	Univariate				Multivariate		
	HR	CI (95%)	*p*-Value	HR		CI (95%)	*p*-Value
**Progression Free survival**							
Age at diagnosis	1.01	(0.99–1.03)	0.474				
BRCA mutated	**0.44**	**(0.28–0.69)**	**<0.001**	**0.36**		**(0.22–0.59)**	**<0.001**
Stage FIGO IV	0.7	(0.43–1.14)	0.156				
CRS score^*^							
CRS2	0.76	(0.47–1.23)	0.267	1.01		(0.55–1.85)	0.970
CRS3	**0.34**	**(0.21–0.57)**	**<0.001**	**0.44**		**(0.23–0.83)**	**0.011**
No Residual tumor (CC0)	**0.5**	**(0.3–0.82)**	**0.007**	**0.29**		**(0.14–0.57)**	**<0.001**
HIPEC yes	1.27	(0.75–2.17)	0.376				
Maintenance Therapy							
Bevacizumab	1.45	(0.87–2.43)	0.153				
ParpI	0.86	(0.5–1.47)	0.584				
Others	1.31	(0.39–4.41)	0.660				
Oligometastatic Recurrence	0.74	(0.47–1.16)	0.184				
**Overall Survival**	**HR**	**CI (95%)**	***p*-Value**	**HR**		**CI (95%)**	***p*-Value**
Age at diagnosis	1.01	(0.99–1.03)	0.514				
BRCA mutated	**0.45**	**(0.27–0.75)**	**0.002**	**0.48**		**(0.28–0.84)**	**0.009**
Stage FIGO IV	0.91	(0.57–1.45)	0.703				
CRS score^*^							
CRS2	0.74	(0.48–1.13)	0.160	1		(0.54–1.88)	0.994
***CRS3***	**0.28**	**(0.17–0.46)**	**<0.001**	**0.33**		**(0.16–0.7)**	**0.004**
No Residual tumor (CC0)	**0.43**	**(0.28–0.67)**	**<0.001**	0.54		(0.29–1.02)	0.058
HIPEC yes	1.51	(0.84–2.68)	0.166				
Maintenance Therapy							
Bevacizumab	0.84	(0.55–1.3)	0.433	1.18		(0.62–2.25)	0.611
ParpI	**0.5**	**(0.3–0.84)**	**0.009**	1.09		(0.52–2.27)	0.822
Others	0.94	(0.29–3.09)	0.925	1.99		(0.25–16.12)	0.520
Oligometastatic Recurrence	0.58	(0.32–1.05)	0.070				

HR: Hazard Ratio; CI: Confidence Interval; VUS: Variant of Unknown Significance; CRS: Chemotherapy Response Score; HIPEC: Hypertermic Intraperitoneal Chemotherapy; CC0: Complete Citoreduction; ParpI (Poly (ADP-ribose) polymerase inhibitors.

*CRS1 as reference.#Bold values indicate statistically significant variables (p < 0.05)

Similar results were obtained regarding OS, CRS3 patients had significantly longer median OS compared to CRS2 and CRS1 (93.9 vs. 37.2 vs. 31.7 months, respectively; *p* < 0.001) (Figure 2). Univariate COX analysis identified CRS3 response (HR = 0.28; 95%CI: 0.17–0.46), BRCA mutation (HR = 0.45; 95%CI: 0.27–0.75), maintenance therapy with PARPi (HR = 0.50; 95%CI: 0.30–0.84), and CC0 resection (HR = 0.43; 95%CI: 0.28–0.67) as significant factors associated with reduce risk of death, while age at diagnosis (HR = 1.01; 95%CI: 0.99–1.03), FIGO IV stage at diagnosis (HR = 0.91; 95%CI: 0.57–1.45), HIPEC use (HR = 1.51; 95%CI: 0.84–2.68), maintenance therapy with Bevacizumab (HR = 0.84; 95%CI: 0.55–1.3) and oligometastatic recurrence (HR = 0.58; 95%CI: 0.32–1.05) were not significant. In multivariate analysis, CRS3 response (HR = 0.33; 95%CI: 0.17–0.70), BRCA mutation (HR = 0.48; 95%CI: 0.28–0.84), and CC0 resection (HR = 0.54; 95%CI: 0.29–1.02) remained independent predictors of reduced risk of death (Table 4). In the sub-analysis of only patients with recurrence, CRS3 patients had significantly longer OS than CRS2 and CRS1 (55.3 vs. 37.2 vs. 31.7 months; *p* = 0.001) (Figure 3). A further sub-analysis of CRS3 patients with oligometastatic disease confirmed the positive impact on OS of surgery or radiotherapy compared to chemotherapy alone (*p* = 0.038) (Supplementary figure 3).

## Discussion

In this retrospective single-center study, CRS3 is associated with an oligometastatic pattern of recurrence in HGSOC treated with NACT and IDS.

### Summary of main results

In our cohort, regardless of the number of NACT cycles, CRS3 represented the largest subgroup (43.3%), reflecting the accuracy of surgical timing for IDS at our center.^4^^,^^21^ Compared to CRS1/2, CRS3 patients showed significantly greater reductions in PCI and Fagotti scores following NACT, achieved higher rates of CC0, and more frequently underwent minimally invasive IDS. Within recurrent patients, 23% developed an oligometastatic pattern, in line with previous literature.^6^^,^^8^ This pattern was more common among CRS3 patients and associated with significantly higher use of SDS (5.18-fold) and radiotherapy (19.2-fold). This translated into a median OS benefit compared to CRS2 and CRS1, respectively, despite only modest differences in PFS of approximately 2 months.

### Results in the context of published literature

The concept of oligometastatic ovarian cancer is gaining traction, supported by trials like DESKTOP III^28^ and MITO RT-03/RAD,^29^ which demonstrated survival benefits from local control, in line with our results. However, predictive tools for recurrence patterns remain limited.^25^^,^^30^ Earlier studies^30^^,^^31^ suggested that compared to PDS, NACT may favor the emergence of resistant subclones, potentially leading to more aggressive recurrence.^9^^,^^32^ However, in a post-hoc analysis of the PRIMA trial, Kamrava et al. reported a 93.2% rate of oligometastatic recurrence among 190 patients without residual disease who were treated with niraparib, with no significant difference in the number of recurrent lesions between the PDS and IDS groups.^33^ In our cohort, PARP inhibitor use was lower (36%), which may explain the lack of statistical significance observed in the logistic regression model (*p* = 0.196). However, it is interesting to note that, although CRS2 and CRS3 patients received similar PARPi exposure (36% vs. 45%, *p* = 0.196), the rate of oligometastatic recurrence was substantially different between the two groups (8.9% vs. 46%, respectively; *p* = 0.001). These findings confirm the PARPi efficacy in controlling residual clones and that its activity is modulated by the underlying tumor biology, for which CRS serves as an early and reproducible indicator: CRS3 tumors, typically platinum-sensitive, respond better to PARPi, while CRS1/2 tumors are more likely to harbor resistant residual clones.^34^^,^^35^ These observations underscore the potential role of CRS score in stratifying patients for maintenance therapy intensity and modality. In this context, the 4-tier stratification risk model proposed by Marchetti et al, incorporating CRS, BRCA/HRD status, and the KELIM score, represents a promising framework for optimizing personalized therapy.^36^ While our study does not directly validate this model, the association between CRS3 and favorable recurrence patterns observed supports the rationale for incorporating CRS into clinical-molecular algorithms.

In their study, CRS score presented linear correlation with KELIM score as expected, while no association was found with BRCA status, consistent with our data. This may lie in the heterogeneity of BRCA mutations, some of which are less platinum-sensitive.^37^ However, PARPi appear effective across BRCA subtypes,^38^ which may explain why Loverro et al., in a fully PARPi-treated cohort,^25^ identified BRCA mutation as a predictor of oligometastatic recurrence, a finding not replicated in our population with limited PARPi use. Finally, in the exploratory analysis of the PRIMA trial, Kamrava et al. found that HRD-positive patients had fewer recurrent lesions than HRD-negative ones, consistent with the known sensitivity of HRD tumors to both platinum and PARPi.^39^ This supports the hypothesis of CRS as a predictor of HRD status as proposed by Wilke et al., who identified CRS3 as a strong predictor of HRD positivity (OR = 4.06; *p* = 0.004),^40^ and in line with our data, in which almost all the CRS3 patients tested for HRD status resulted positive compared to the CRS1/2 patients (94% vs 50% vs 60% respectively). Conversely, Marchetti et al. reported only partial overlap, with 45.9% of CRS3 patients being HRD-positive. Given the limited number of patients tested for HRD in our study, these findings must be interpreted with caution and considered purely exploratory. While our data suggest a potential link between pathologic response and molecular status, this remains a hypothesis-generating observation that warrants further prospective validation in larger tested cohorts.

### Strengths and weaknesses

The main limitation of this study is the lack of comprehensive HRD status data. HRD testing was implemented only in the later years of the inclusion period, resulting in data availability for just 15.9% of patients. Given that HRD status is central to modulating chemosensitivity and determining the benefit of PARP inhibitors, its absence limits the molecular context of our findings regarding recurrence.

Additionally, excluding patients deemed ineligible for IDS due to poor NACT response may have introduced selection bias toward more chemo sensitive tumors, may explain the higher percentage of CRS3 patients (43.3%) compared to previous studies.^34^^,^^40^ Consequently, these results apply primarily to patients eligible for surgery following NACT and cannot be extrapolated to the entire population of patients treated with NACT. Furthermore, the study population is characterized by heterogeneity in neoadjuvant regimens and surgical strategies over time, which may have influenced the results presented. However, this is a limitation of NACT studies, where surgical eligibility is driven by cytoreductive potential. As a retrospective single-center analysis, the study carries the usual risks of confounding and selection bias. Nonetheless, it benefits from a well-defined cohort treated in a high-volume tertiary center, with consistent pathological assessment by a dedicated gynecologic oncology pathologist.

### Implications for practice and future research

CRS represents a powerful early biomarker with significant implications for recurrence stratification and treatment tailoring. Beyond its role as a predictor of oligometastatic recurrence and improved overall survival, the CRS score serves as a critical bridge between molecular status and the clinical world. Specifically, the association between CRS3, higher CC0 rates, and the increased feasibility of minimally invasive surgery reported in this study, supports a more tailored surgical approach for patients achieving a complete clinical response after NACT, as previously reported^41^^,^^42^ However, several critical questions remain unanswered. First, baseline clinical predictors of CRS status are lacking. Second, it is unclear whether CRS3 patients require additional chemotherapy after IDS or can proceed directly to maintenance. Third, CRS1/2 tumors may benefit from intensified maintenance strategies, including antibody-drug conjugates, as recent data suggest up to 55% of OC co-express multiple targets, as reported by Hamagawa et al. at ESMO 2025.^43^

## Conclusions

CRS3 is correlated with a recurrence pattern more amenable to secondary locoregional strategies such as surgery or radiotherapy. These findings support the potential role of CRS not only as a prognostic indicator but also as a tool for tailoring surveillance and maintenance approaches, particularly when associated with molecular data. Future prospective and externally validated studies are warranted.

## Supplementary Material

oyag055_Supplementary_Data

## Data Availability

The data of this study are available from the corresponding author upon reasonable request to the corresponding author at: https://zenodo.org/records/15880187.
