# Supplementary material for: Association between pathologic chemotherapy response score and pattern of recurrence in advanced high-grade serous ovarian cancer
Source: Oncologist. 2026 Mar 30;31(4):oyag055. doi: 10.1093/oncolo/oyag055 (PMC13049597; doi:10.1093/oncolo/oyag055)
Supplement: oyag055_Supplementary_Data [file oyag055_supplementary_data.zip › Supplementary table.docx]

| Supplementary Table 1: Posthoc analysis and paired Bonferroni correction | | | | |  |
| --- | --- | --- | --- | --- | --- |
| **Variable** | **Group 1** | **VS** | **Group 2** | **p_value** |  |
| Age at diagnosis | CRS2 |  | CRS1 | 0.208 |  |
|  | CRS3 |  | CRS1 | 1.000 |  |
|  | CRS3 |  | CRS2 | 0.575 |  |
| BRCA status | CRS1 |  | CRS2 | 1.000 |  |
|  | CRS1 |  | CRS3 | 1.000 |  |
|  | CRS2 |  | CRS3 | 0.848 |  |
| HRD status | CRS1 |  | CRS2 | 1.000 |  |
|  | CRS1 |  | CRS3 | 0.647 |  |
|  | CRS2 |  | CRS3 | 0.078 |  |
| BMI | CRS2 |  | CRS1 | 1.000 |  |
|  | CRS3 |  | CRS1 | 1.000 |  |
|  | CRS3 |  | CRS2 | 1.000 |  |
| Baseline CA125 (U/ml) | CRS2 |  | CRS1 | 1.000 |  |
|  | CRS3 |  | CRS1 | 1.000 |  |
|  | CRS3 |  | CRS2 | 1.000 |  |
| Baseline PCI | CRS2 |  | CRS1 | 0.131 |  |
|  | CRS3 |  | CRS1 | **0.030** |  |
|  | CRS3 |  | CRS2 | 1.000 |  |
| Baseline Fagotti Score | CRS2 |  | CRS1 | 1.000 |  |
|  | CRS3 |  | CRS1 | 1.000 |  |
|  | CRS3 |  | CRS2 | 1.000 |  |
| Stage at diagnosis (III/IV) | CRS2 |  | CRS1 | 1.000 |  |
|  | CRS3 |  | CRS1 | 1.000 |  |
|  | CRS3 |  | CRS2 | 1.000 |  |
| Numbers of NACT cycles | CRS2 |  | CRS1 | 1.000 |  |
|  | CRS3 |  | CRS1 | 1.000 |  |
|  | CRS3 |  | CRS2 | 1.000 |  |
| PCI post NACT | CRS2 |  | CRS1 | **0.000** |  |
|  | CRS3 |  | CRS1 | **0.000** |  |
|  | CRS3 |  | CRS2 | **0.000** |  |
| Fagotti score post NACT | CRS1 |  | CRS2 | 1.000 |  |
|  | CRS1 |  | CRS3 | 1.000 |  |
|  | CRS2 |  | CRS3 | 1.000 |  |
| Type of IDS | CRS1 |  | CRS2 | 1.000 |  |
|  | CRS1 |  | CRS3 | **0.030** |  |
|  | CRS2 |  | CRS3 | **0.002** |  |
| Surgery timing, minutes | CRS2 |  | CRS1 | 1.000 |  |
|  | CRS3 |  | CRS1 | 0.506 |  |
|  | CRS3 |  | CRS2 | 0.442 |  |
| Aletti Complexity score | CRS2 |  | CRS1 | 0.369 |  |
|  | CRS3 |  | CRS1 | **0.001** |  |
|  | CRS3 |  | CRS2 | **0.008** |  |
| HIPEC use | CRS2 |  | CRS1 | 0.587 |  |
|  | CRS3 |  | CRS1 | 0.808 |  |
|  | CRS3 |  | CRS2 | 1.000 |  |
| Residual Tumor | CRS2 |  | CRS1 | **0.013** |  |
|  | CRS3 |  | CRS1 | **0.000** |  |
|  | CRS3 |  | CRS2 | **0.039** |  |
| Maintenance Therapy | CRS1 |  | CRS2 | **0.004** |  |
|  | CRS1 |  | CRS3 | **0.004** |  |
|  | CRS2 |  | CRS3 | 0.196 |  |
| Type of recurrence (any site) (Oligo vs Multi) | CRS1 |  | CRS2 | 1.000 |  |
|  | CRS1 |  | CRS3 | 0.069 |  |
|  | CRS2 |  | CRS3 | **0.001** |  |
| Peritoneal recurrence  (Oligo vs Multi vs No lesions) | CRS1 |  | CRS2 | 1.000 |  |
|  | CRS1 |  | CRS3 | **0.033** |  |
|  | CRS2 |  | CRS3 | **0.003** |  |
| Nodal recurrence  (Oligo vs Multi vs No lesions) | CRS1 |  | CRS2 | 0.945 |  |
|  | CRS1 |  | CRS3 | 0.530 |  |
|  | CRS2 |  | CRS3 | 0.094 |  |
| Nodal recurrence  (Oligo vs Multi vs No lesions) | CRS1 |  | CRS2 | 0.720 |  |
|  | CRS1 |  | CRS3 | 1.000 |  |
|  | CRS2 |  | CRS3 | 0.553 |  |
| 1Median (Q1, Q3); n (%); 2Kruskal-Wallis rank sum test; Fisher's exact test; Pearson's Chi-squared test; One-way analysis of means. CRS: Chemotherapy Response Score; IDS: Interval Debulking surgery; HIPEC: Hyperthermic Intraperitoneal Chemotherapy; CC0: Complete Citoreduction; CC1: Incomplete Citoreduction; ParpI (Poly (ADP-ribose) polymerase inhibitors. | | | | | |

| Supplementary Table 2: Distribution of CRS according to number of NACT cycles | | | | |
| --- | --- | --- | --- | --- |
| **Variable** |  | **3-4 cycles**  **N = 93^1^** | **> 5 cycles** **N = 145^1^** | **p-value^2^** |
| CRS |  |  |  | 0.820 |
| CRS1 |  | 18 (19%) | 25 (17%) |  |
| CRS2 |  | 37 (40%) | 55 (38%) |  |
| CRS3 |  | 38 (41%) | 65 (45%) |  |
| Oligometastatic Recurrence |  | 9 (19%) | 16 (26%) | 0.413 |
| 1Median (Q1, Q3); n (%); 2Kruskal-Wallis rank sum test; Fisher's exact test; Pearson's Chi-squared test; One-way analysis of means. CRS: Chemotherapy Response Score; NACT: Neoadjuvant Chemotherapy; N: number | | | | |
|  | | | | |
