# Supplementary material for: Association between pathologic chemotherapy response score and pattern of recurrence in advanced high-grade serous ovarian cancer
Source: Oncologist. 2026 Mar 30;31(4):oyag055. doi: 10.1093/oncolo/oyag055 (PMC13049597; doi:10.1093/oncolo/oyag055)
Supplement: oyag055_Supplementary_Data [file oyag055_supplementary_data.zip › supplementary figure legend.docx]

**Supplementary Figure Legends**

FIGURE S1: PRISMA Flow diagram;

FIGURE S2: Reverse Kaplan Meier Curve for Follow up timing;

FIGURE S3: Kaplan Meier Curve of CRS3 patients treated with CHT alone vs CRS3 patients treated with combined approach (CHT+Surgery/Radiotherapy)
