# Supplementary figures and images for: Association between pathologic chemotherapy response score and pattern of recurrence in advanced high-grade serous ovarian cancer
Source: Oncologist. 2026 Mar 30;31(4):oyag055. doi: 10.1093/oncolo/oyag055 (PMC13049597; doi:10.1093/oncolo/oyag055)

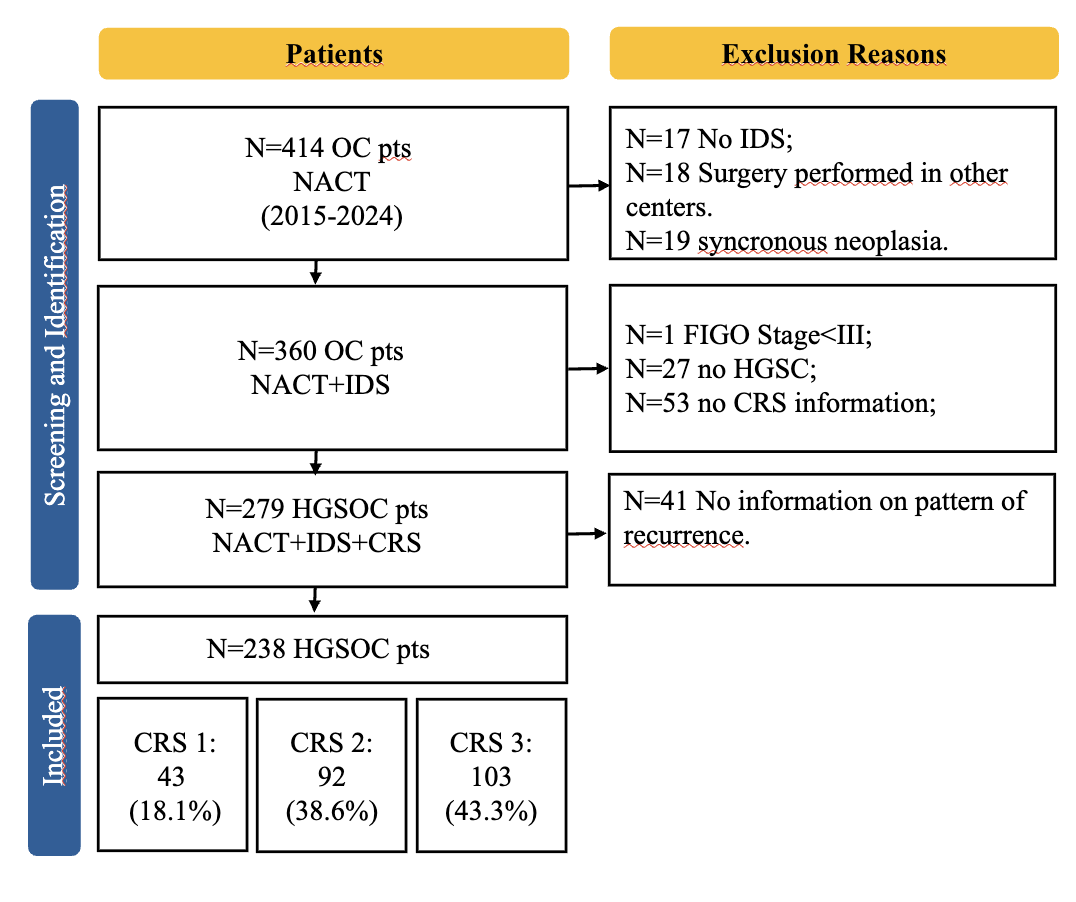

Supplement: oyag055_Supplementary_Data [file oyag055_supplementary_data.zip › Supplementary Figure 1.png]

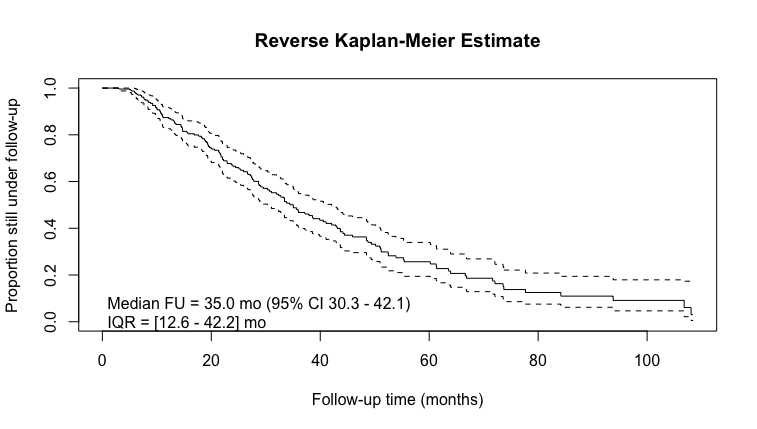

Supplement: oyag055_Supplementary_Data [file oyag055_supplementary_data.zip › Supplementary Figure 2.png]

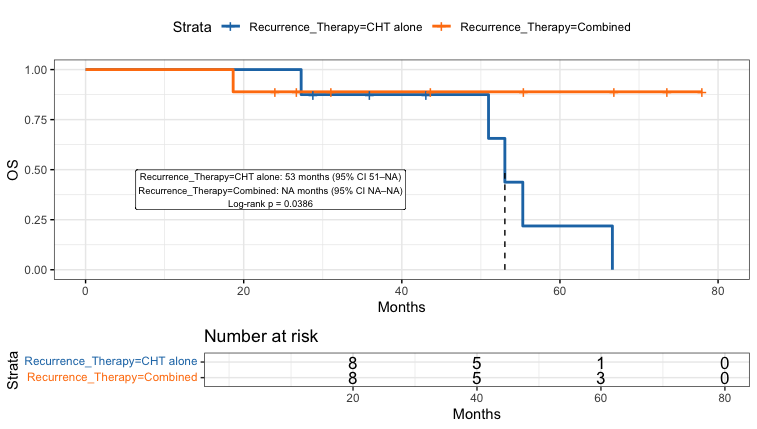

Supplement: oyag055_Supplementary_Data [file oyag055_supplementary_data.zip › Supplementary Figure 3.png]
